# Dermotoscope assisted diagnosis of adolescent bullous pilomatricoma: six case reports

**DOI:** 10.1186/s12887-024-04914-9

**Published:** 2024-07-09

**Authors:** Junru Liu, Xiaojie Liu, Yan Qu, Shuqing Zhang

**Affiliations:** 1 Department of Dermatology, Laishan Branch of Yantai Yuhuangding Hospital, Shuanghexi Road 59, Yantai, Shandong China; 2 Department of Dermatology, Tianjin Academy of Traditional Chinese Medicine Affiliated Hospital, Tianjin, China

**Keywords:** Pilomatricoma, Dermotoscope, Bullous variant, Case report

## Abstract

**Background:**

Pilomatricoma (PM) is a cutaneous benign neoplasm derived from the hair matrix. It clinically presents as a solitary and firm nodule overlying normal epidermis and is usually not easy to be noticed at early stage. Nevertheless, when special bullous lesion occurs in a short time or even ulcerates, preoperative diagnosis by a dermatologist is often challenging especially when the pediatric patients refuse biopsy.

**Case presentation:**

We present six bullous PM cases and particularly conduct correlation analysis on the dermotoscopy and histopathology detection data. The basic information, medical history, symptoms and lesion morphology results of the patients were also provided. We found that the incidence of bullous PM was higher in females than in males, and most patients were adolescents and the predilection location seem to be consistent in the vaccine injection site. The dermatoscopic features of bullous PM reported were luminous yellow structure below, with gray-blue homogeneous areas and branched capillary. The histological features were consistent with PM, and evident epidermis bullae were above the tumor with extraordinary dilation of lymphangion in the upper dermis. The patients described in this study were Chinese patients in Han population included 4 females and 2 males, coincidentally, they are almost teen-age, respectively are 5,11,17,19,21,22 year-old.

**Conclusions:**

This study reported and analyzed the dermotoscopy and clinical characteristics of bullous PM, dermotoscopy may guide as a rapid and reliable technique in bullous PM diagnosis.

## Background

Bullous pilomatricoma (bullous PM), known as anetodermic pilomatricoma or lymphangiectatic pilomatricoma, is a particular type of lymphangiectatic pilomatricoma with a bullous appearance. At present, the diagnosis of PM mainly depends on histopathological examination. Although PM has typical pathological features [1], but in clinical practice, there’s also some difficult to distinguish from other tumors such as trichoepithelioma, trichoblastoma and calcinosis cutis. Currently, there is a lack of effective preoperative examination for PM except the biopsy, but in recent investigations, different noninvasive diagnostic tests have been tried, including MRI, high-frequency ultrasonography. Dermotoscopy was first applied by dermatologists on the PM in 2018 [2] P. Huet et al. have summarized the main dermatoscopic features of PM, but reports about the bullous variant are still scarce [3]. Therefore, we presented a total of six cases of bullous variant, and further investigated the dermatoscopic features and corresponding pathological changes of this PM variant based on previous research.

## Case presentation

**Fig. 1:**
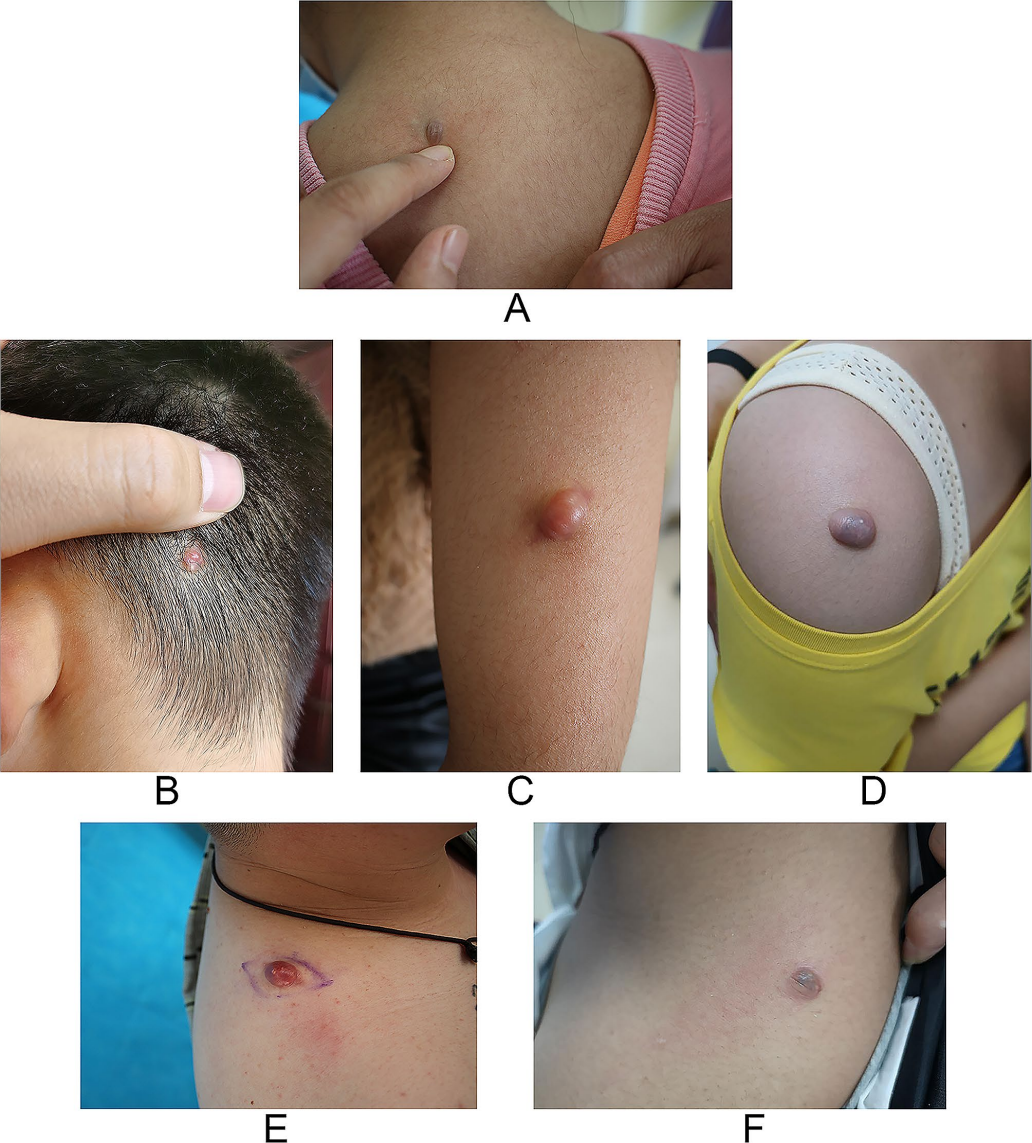
(**A-F**) Clinical manifestation of 6 bullous PM

From 2019 to 2021, six patients were diagnosed with bullous PM and treated in Laishan Branch of Yantai Yuhuangding Hospital and Tianjin Academy of Traditional Chinese Medicine Affiliated Hospital. The medical records, dermatoscopic and histopathological examination results of the six cases were retrospectively analyzed. Quantitative analysis was performed for the measurement data on tumor and the clinical information of the patients (Table 1). The clinical manifestations are shown in Fig. 1A-F. No abnormity was found in the past medical history, vaccination history and family medical history of the patients. All patients visited the dermatology department for the first time, and none of them received any other treatments before. Several patients had occasional pain symptoms. Tumor size was proportionate to the tumor course and all bullae erupted in a short time. Dermotoscopy detection was applied using **DERMAT II** (Dermat com, Beijing) and **DL3** (Dermlite com, America), with the immersion and polarized mode for inspection and randomly adjusted exposure intensity. Histological examination was performed in the pathology departments of both hospitals, and tumor tissue sections were stained with HE and then read by both pathologists and dermatologists. All patients underwent invasive surgery with minimally small incisions, and no recurrence occurred after two years of follow-up, all patient guardians expressed great satisfaction for the rapid preoperative diagnosis progress and treatment effect.

**Table 1:** Six patients information and tumor characteristics

Characteristics	Case 1	Case 2	Case 3	Case 4	Case 5	Case 6
**Age**	11	5	21	19	22	17
**Sex**	female	male	female	female	male	female
**Course of Tumor (month)**	6	4	12	14	6	9
**Course of Bullous eruption (weeks)**	2	1	2	3	4	1
**Tumor Position**	Left shoulder	Left occipitalia	Left upper arm	Left upper arm	Right upper arm	Left upper arm
**Tumor Size (cm)**	1.5 × 1.6 × 0.6	1.3 × 0.7 × 0.5	2.5 × 1.8 × 0.7	1.9 × 1.5 × 0.9	2.1 × 1.2 × 0.8	2.2 × 1.2 × 0.7
**Symptom**	No	Slight pruritus	Slight pain	No	Slight pain	No
**Dermotoscopic Characteristics**						
**foggy structure**	+	+	+	+	+	+
**Luminous yellow lump structure**	+	+	+	+	+	+
**Arborizing vascular pattern**	+	+	-	+	+	+
**Circumscribed bluish-grey region**	+	+	+	+	+	+
**White streaks**	-	+	-	+	+	+

Note: The table showed summarizing PM clinical information of the patients, including the epidemiological characteristics and disease duration, clinical symptom and the typical dermotoscopic manifestations

**Abbreviations**: +/- indicates the presence or absence

### Dermatoscopic images presentation

**Fig. 2:**
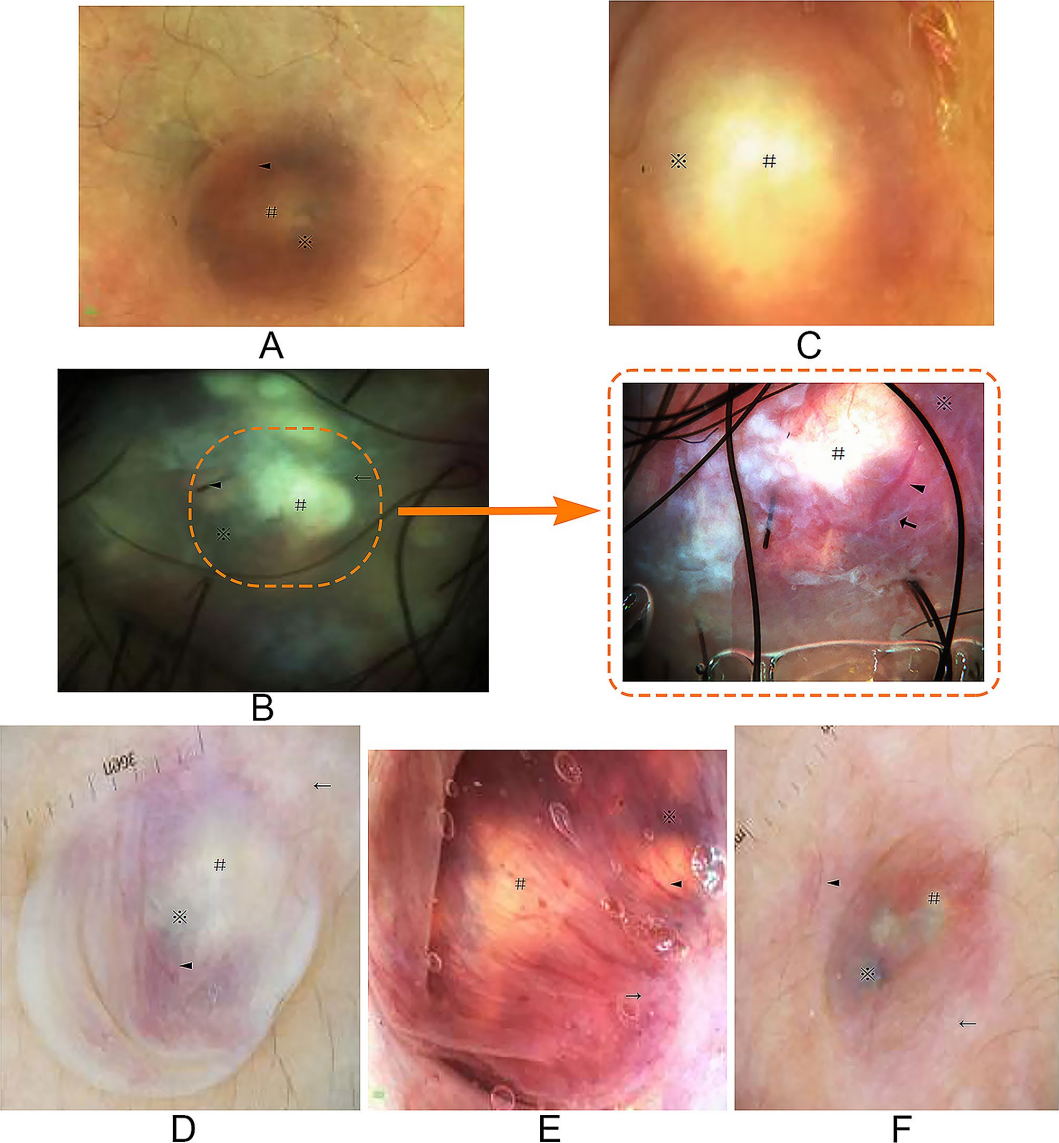
(**A-F**) Dermotoscopic characteristics of 6 bullous PM ×20 # Luminous yellow lump-like structure, ※ Circumscribed Bluish-grey region, ← white streaks, ◄ branching vessels; 2B×40: more high magnification dermoscopic characteristics of case2 ←white streaks, ◄ branch capillary

The dermatoscopic images of the six cases **(**Fig. 2A-F**)** showed foggy and luminous yellow structure below, with gray-blue homogeneous areas surrounding yellow structure. Overexposure could occur when we increased polarized light brightness, and then the gloss of the yellow structure gave a high refraction, cluing its texture is dense. Branched or linear capillaries were scattered or circumferentially distributed on the surface of the tumor or around after enlarging the dermotoscopy lens **(**Fig. 2B**)**. White string streaks could also be found in some patients.

### Histopathology appearances

**Fig. 3:**
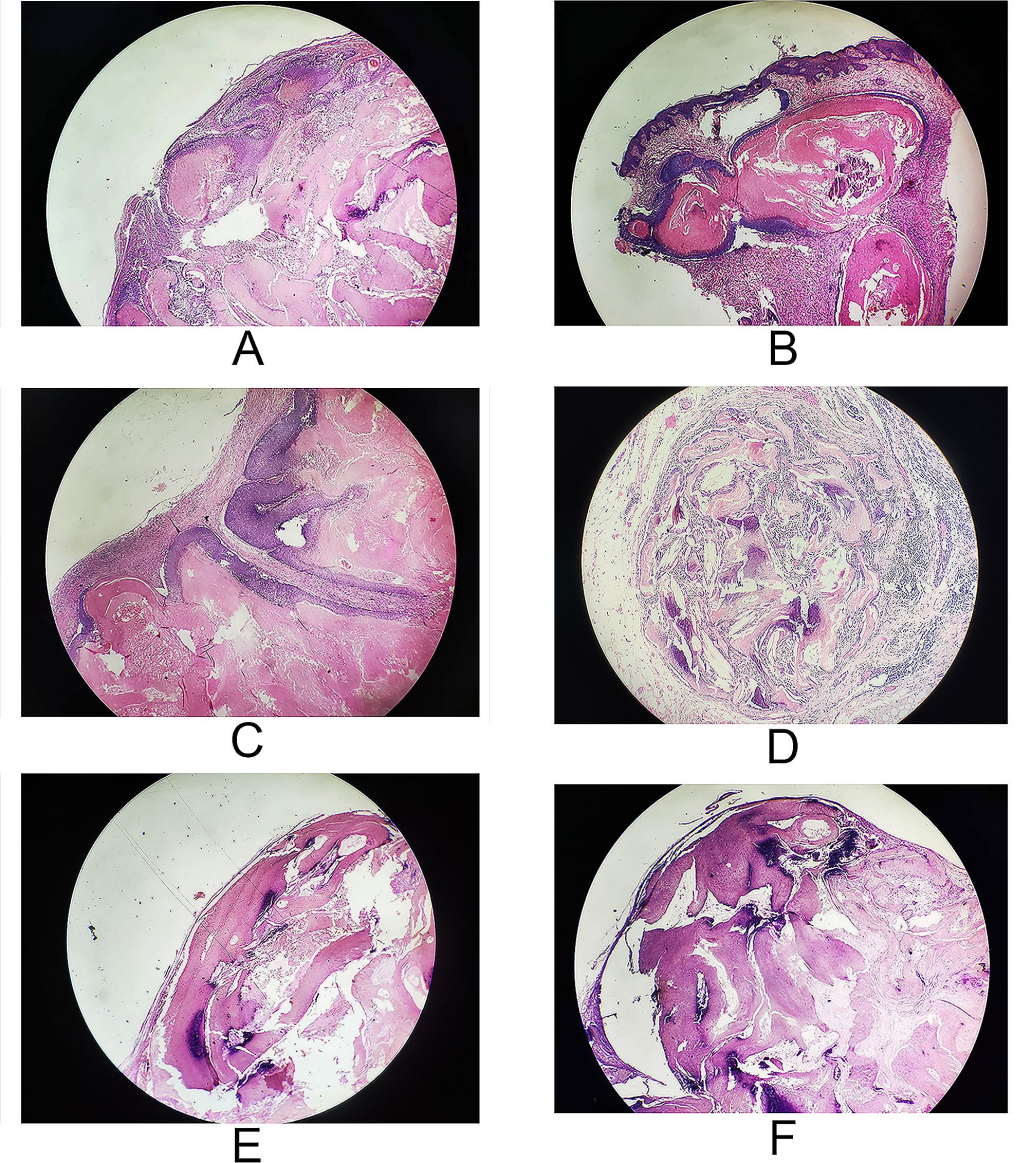
(**A-F**) Pathological features of 6 bullous PM ×40 included angiotelectasis and lymphangiectasia, basaloid and ghost cells, focal calcifications

All tumor histological examinations showed almost identical characteristics **(**Fig. 3A-F**)** as subepidermal fissure, mildly edema dermis, sparse collagen fibers, angiotelectasis and lymphangiectasia inside the dermal papillary layer, deeper in the dermis, multilobulated tumor, surrounded by a fibrous pseudo-capsule of compressed connective tissue. The admixture of basaloid and ghost cells was seen within the tumor and centrally located calcification was present, histocyte and phagocytic giant cell reaction was diffused.

## Discussion and conclusions

PM also refers to pilomatrixoma and calcifying epithelioma of malherbe, and it is a type of benign dermal-subcutaneous tumor derived from the matrix of the hair follicle [4]. Clinical types of the tumor include familial, perforating, multinodular [5], exophytic, anetodermic, and giant PM [6]. The bullous type is seen in only 3 to 6% of cases [7]. It is seen more frequently in women and children, and often occurs on the shoulders and upper arms. Two theories have been proposed to explain its pathogenesis, and one theory states that the growth of the tumor may cause obstruction of lymphangion with subsequent leakage of lymphatic fluid and edema of the dermis. Multiple pilomatricoma have been associated with many disorders, such as Turner syndrome, Gardner syndrome, sarcoidosis, and the neuromuscular disorder myotonic dystrophy. Therefore, lower calcium levels are believed to cause high cell proliferation but lower terminal differentiation, and possibly lead to shadow or ghost cells, which are observed as a histopathologic feature in pilomatricomas [8]. Clinically, bullous PM needs to be differentiated from lymphangioma, hydrocystoma, epidermal cyst, etc. [9]. Reviewing previous literature reports [10], we found the evidence about the inducement of bullous PM, which was associated with the development of anetodermic PM with mechanical trauma. Therefore, we speculated that this specific pathogenic site might not be a coincidence, and perhaps there has a possible pathogenic mechanism related to the vaccination on the left side, this conjecture also was validated by recent case reports [11–13]. Clinicians should be aware that bullous pilomatricoma may occur after vaccination. In this study, preoperative dermotoscopy examination showed that bullous PM had characteristic changes, which mainly manifested as: (1) the epidermal structure with good transmittance and the foggy structure presented together with blisters; (2) the luminous yellow lump-like structure of high refraction below the epidermis under polarized light; (3) circumscribed the yellow gray-blue homogeneous region. In addition, the large branching capillaries are scattered on the surface of tumor vesicles with a relatively short course of disease, but no obvious blood vessels are seen on the surface of tumor vesicles with a relatively long course of disease. However, dilated branching capillaries can be found in and around the tumor base.

The dermotoscopy characteristics of PM were first discovered by Pedro Zaballos in 2008. With the improvement of relevant clinical research, its characteristics are gradually revealed in more details in previous reports on different types of PM, and the dermatoscopic features included yellowish-white structures with white streaks, reddish homogeneous areas, and polymorphous vessels and blue-grey structureless areas [14]. The key update in our report is that as a rare and special clinical type, bullous PM has extremely thin-walled bulla-like surface so that the polarized light can be transmitted to the full extent to discover more typical dermatoscopic structure of the tumor, thus strengthening the corresponding relationship between dermatoscopic structure and pathological structure. Based upon the dermatoscopic manifestations and pathological changes, we speculated that the high-refractive yellow lump-like structure, gray-blue region and branching blood vessel are corresponded to the block-like calcification tissue and shadow (ghost) cells and the basophils surrounding shadow cells in histopathological changes. The large branching vascular structure is indispensable for the differentiation of epidermoid cysts [15], and the high-refractive mass structure is also different from the yellow lobular structure of steatadenoma [16]. The gray-blue structureless areas of bullous PM is essentially different from the dermatoscopic appearances of bluish gray/black globules structure of basal cell carcinoma [17]. The blue-gray globules structure in basal cell carcinoma is corresponded to basal-like tumor cells that are arranged in a palisade pattern, and there is a tissue contraction gap around them. Therefore, dermoscopy shows isolated blue-gray globules with more sharp edges [18]. The dermoscopy provides more apparent evidence to support diagnosis and has more advantages over other noninvasive preoperative detection methods such as MRI and ultrasound. Accurate preopretive dignosis is helpful to patients acccept surgical procedures while surgeon have confidence to develop a minimally invasive surgical protocols [19], thus, excellent aesthetic results are obtained. Our study can enhance the understanding of the tumor nature and inducement of pilomatricoma, thereby assisting clinicians in the development of minimally invasive surgical plans.

In conclusion, the dermatoscopic features of bullous PM are specific, and the most prominent feature is the high refractive luminous yellow lump-like structure, accompanied by the nodules of hard texture in palpation. Dermotoscope, as a noninvasive examination, is more rapid, economic and valuable diagnostic tool for this special type pilomatricoma.

## Data Availability

No datasets were generated or analysed during the current study.

## Abbreviations

PM: pilomaricoma
HE: hematoxylin-eosin
